# Association of adult attachment styles and early traumas with suicidal behaviour

**DOI:** 10.1192/j.eurpsy.2025.1327

**Published:** 2025-08-26

**Authors:** N. M. Szeifert, B. Olah, X. Gonda

**Affiliations:** 1 Department of Sports Medicine, Semmelweis University; 2 Doctoral School of Psychology, ELTE Eotvos Lorand University, Budapest; 3 Department of Behavioural Sciences, University of Debrecen, Faculty of Medicine; 4 Health Care Service Units, Clinical Psychology Center of CC, University of Debrecen, Clinical Centre, Debrecen; 5 Department of Psychiatry and Psychotherapy, Semmelweis University, Budapest, Hungary

## Abstract

**Introduction:**

Attachment styles formed in early childhood can influence mental health across the lifespan. Secure attachment often correlates with resilience, while insecure or disorganized attachment styles may increase vulnerability to mental health issues such as traumas during formative years and can disrupt typical emotional and psychological development, increasing the risk of later mental health issues, including suicide. Early trauma can influence the impact of adult attachment on suicidal behavior. For instance, people with high early trauma levels may experience heightened sensitivity to attachment-related stressors, increasing suicide risk.

**Objectives:**

Our aim was to analyse the role of adult attachment styles and early traumas in suicidal behaviour, focusing on the potential mediating effect of attachment style in the relationship of early traumas and suicidal behavior.

**Methods:**

357 subjects, 18-85 years of age (mean 37.02 SD=12.86), 33.6% male and 66.4% female. 146 with suicide history, 154 clinical sample without suicide history and 57 non-clinical sample we included in the study. The Adult Attachment Scale (AAS), the Childhood Trauma Questionnaire (CTQ) and the Early Trauma Inventory Self Report Short Form (ETISR-SF) were used. To model the relationship between the variables logistic regression, generalized linear models and mediation analysis were conducted. All models were adjusted for basic demographics.

**Results:**

Severity of emotional abuse (OR=1.064, p=0.004) and neglect (OR=1.064, p=0.007) and the total severity of traumatization by CTQ (OR=1.021, p=0.006) significantly predicted higher risk for suicidal behavior. Higher levels of secure attachment style predicted lower risk of suicide attempt (OR=-0.091, p=0.004). Secure attachment style significantly mediated part of the total effect of early traumatization severity on suicidal behavior (indirect effect=0.0032, p<0.05, Pm=16.5%). We also modeled the relationship between early traumas and attachment style and found multiple associations. For avoidant attachment, significant associations were found with total traumatization scores (ETI: B=0.337, p<0.001; CTQ: B=0.866, p<0.001) and specific adversities, including emotional abuse (ETI: B=2.703, CTQ: B=0.524), emotional neglect, physical abuse, and physical neglect (B=0.469–0.949, all p<0.001). Anxious-ambivalent attachment was linked to total ETI and CTQ scores (B=0.375, 0.088 all p<0.001), emotional abuse (ETI: B=1.605, CTQ: B=0.388), and physical neglect (B=0.261, all p<0.001). Lower secure attachment was associated with CTQ scores for overall traumatization, emotional, and sexual abuse (B=-0.039 to -0.163, all p<0.05).

**Conclusions:**

Our results contributes to understanding how attachment styles and early traumas influence suicidal behavior. Addressing these factors in treatment can lead to personalized and effective interventions, potentially reducing suicide risk among vulnerable individuals.

**Disclosure of Interest:**

None Declared

